# MHC-independent αβT cells: Lessons learned about thymic selection and MHC-restriction

**DOI:** 10.3389/fimmu.2022.953160

**Published:** 2022-07-14

**Authors:** François Van Laethem, Abhisek Bhattacharya, Marco Craveiro, Jinghua Lu, Peter D. Sun, Alfred Singer

**Affiliations:** ^1^ Lymphocyte Development Section, Experimental Immunology Branch, National Cancer Institute, National Institutes of Health, Bethesda, MD, United States; ^2^ Department of Biological Hematology, Centre Hospitalier Universitaire (CHU) Montpellier, Montpellier, France; ^3^ Structural Immunology Section, Laboratory of Immunogenetics, National Institute of Allergy and Infectious Diseases, National Institutes of Health, Rockville, MD, United States

**Keywords:** thymic selection, MHC restriction, T cell receptor, tyrosine kinases, Lck, coreceptors, T cell repertoire

## Abstract

Understanding the generation of an MHC-restricted T cell repertoire is the cornerstone of modern T cell immunology. The unique ability of αβT cells to only recognize peptide antigens presented by MHC molecules but not conformational antigens is referred to as MHC restriction. How MHC restriction is imposed on a very large T cell receptor (TCR) repertoire is still heavily debated. We recently proposed the selection model, which posits that newly re-arranged TCRs can structurally recognize a wide variety of antigens, ranging from peptides presented by MHC molecules to native proteins like cell surface markers. However, on a molecular level, the sequestration of the essential tyrosine kinase Lck by the coreceptors CD4 and CD8 allows only MHC-restricted TCRs to signal. In the absence of Lck sequestration, MHC-independent TCRs can signal and instruct the generation of mature αβT cells that can recognize native protein ligands. The selection model thus explains how only MHC-restricted TCRs can signal and survive thymic selection. In this review, we will discuss the genetic evidence that led to our selection model. We will summarize the selection mechanism and structural properties of MHC-independent TCRs and further discuss the various non-MHC ligands we have identified.

## Introduction

Adaptive immunity depends on the ability of T lymphocytes to recognize foreign antigens. The last three decades have brought tremendous insight into the antigen recognition properties of αβT cells. Experiments performed by Zinkernagel and Doherty more than forty years ago documented the ability of αβT cells from lymphocytic choriomeningitis virus (LCMV)-infected mice to kill *in vitro* LCMV-infected target cells only if the T cells and the target cells shared at least one H-2 antigen (1–3). Their observations and interpretation led them to their 1996 Nobel Prize award for discovering major histocompatibility complex (MHC) restricted antigen recognition, now a well-established T cell immunology hallmark (4, 5). The simultaneous recognition of antigenic peptides with self-MHC molecules highlights a unique receptor-ligand interaction that is unparalleled in biology. A fragile balance in this unusual interplay is required to control T cell immunity, providing effective protection from infection while avoiding T cell mediated autoimmunity.

Both T and B lymphocytes use the same gene reco0mbination machinery to create their antigen receptor diversity, but those receptors recognize their ligands in fundamentally different ways (6). Antibodies generated by B cells recognize a wide array of three-dimensional epitopes on native antigenic proteins or glycolipids (7). Somatic recombination of the TCR loci generates tremendous diversity, but αβTCRs focus only on foreign and self-peptides presented by self MHC molecules (8). Thymocytes rearrange genomic regions on both TCRα and TCRβ loci, generating in the process diverse *de novo* segments called complementarity determining regions 3 (CDR3) that are responsible for peptide recognition (9, 10), with diversity being further enhanced by random addition and deletion of nucleotides. The other two regions, CDR1 and CDR2, are germline-encoded and carry limited diversity which is encoded in the variable domains of both α and β TCR chains (11).

On the other side of the equation, MHC proteins present peptides to T cells to discriminate between self and non-self. Immune evasion by pathogens is rendered more difficult by two major characteristics of the MHC loci. First, the MHC is polygenic and contains several different MHC-I and MHC-II genes so that each individual possesses a set of MHC molecules with different ranges of peptide-binding specificities. Second, the MHC genes show the greatest degree of polymorphism in the human genome (12). Multiple variants of the same gene exist within the population as a whole and therefore the extent of peptides presented to T cells is virtually unlimited. This heterogeneity of MHC alleles at the individual and population levels provides the immune system a robust mechanism to counteract pathogens evading MHC presentation and T cell responses.

During T cell development in the thymus, positive and negative selection allow immature thymocytes to be screened for ligand specificity. To survive selection and undergo differentiation, thymocytes must express TCRs that engage intra-thymic ligands and successfully generate intracellular signals. This process is crucial for thymic selection, as the vast majority of T cell precursors bear “useless” TCRs that are incapable of producing signals and therefore undergo death by neglect.

**Figure 1:**
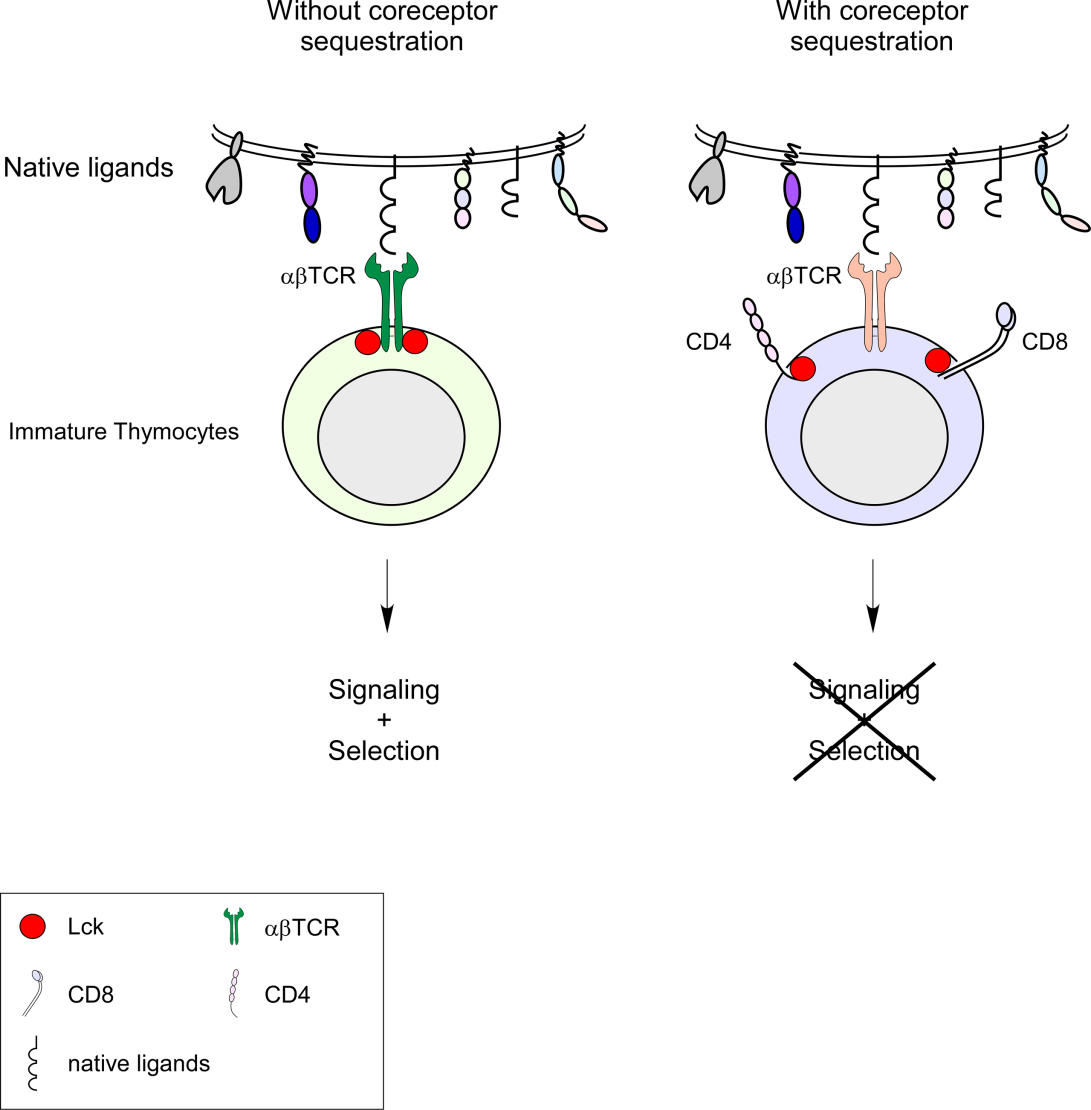
Selection model and MHC-independent αβT cell selection. In immature DP thymocytes, the protein kinase Lck is sequestered by the CD4 and CD8 coreceptors. In the presence of coreceptors (right side of panel), MHC-independent αβTCRs cannot receive any Lck-mediated signaling and therefore cannot be selected. In the absence of coreceptors (left side of the panel), MHC-independent αβTCRs have access to Lck (coreceptor-free and TCR-associated) and can be signaled and selected. Lck sequestration by CD4 and CD8 coreceptors ensures that only MHC-specific αβTCRs can be signaled and selected.

A few years ago, we proposed the selection model to describe the molecular basis of MHC restriction (13). In the selection model, nothing intrinsic to the TCR structure imposes MHC restriction on the randomly generated αβT cell repertoire (**Figure 1**). Like antibodies generated by the same recombination machinery, the pre-selection αβTCR repertoire can recognize a wide variety of antigens, including MHC and non-MHC ligands but only MHC-restricted αβTCRs can signal in the thymus. The TCR itself does not possess intrinsic signaling capabilities but requires the co-engagement of coreceptors to initiate signaling. TCR ligation leads to the tyrosine phosphorylation within immunoreceptor tyrosine-based activation motifs (ITAMs) on all TCR-associated CD3 chains (14). This phosphorylation is carried out by the tyrosine kinases of the Src family of kinases, i.e. Lck and Fyn. Subsequently, another tyrosine kinase, ZAP-70, is recruited to the TCR/CD3 complex, where it binds the phosphorylated ITAMs and can now be phosphorylated and activated by Lck. The adaptor proteins LAT and SLP-76 are then phosphorylated by active ZAP-70 and recruit mediators to propagate downstream signaling pathways. Additionally, signaling initiation is strictly dependent on coreceptor binding to its specific MHC.

Thus, the fact that coreceptors only recognize MHC ligands invokes the hypothesis that MHC restriction is directly imposed by the TCR signaling requirements for thymic selection (**Figure 1**).

After recalling fundamental aspects of early TCR signaling, we will review experimental evidence in favor of the selection model of MHC restriction.

## LCK and TCR signaling

Lck is a member of the Src family of protein tyrosine kinases first identified in the 1980s and plays a crucial role in initiating the TCR signaling cascade (15, 16). Lck is critically important during T cell development and T cell activation. Germline Lck-deficient mice or immune-deficient patients with Lck mutations show profound T cell developmental defects (17, 18). The function of Lck and its conformational state are regulated by several tyrosine kinases and phosphatases acting on its phosphorylation status (19, 20). The phosphorylation of the activating tyrosine (Y394) in the catalytic domain results in an open conformation of Lck and therefore induces its kinase activity, whereas the phosphorylation of tyrosine (Y505) at the C-terminal domain is thought to induce a closed conformation and therefore inhibits Lck activity (21). Collectively, the activity of Lck is tightly regulated by a great number of biochemical modifications, conformational changes and signaling circuits. These complicated regulatory mechanisms highlight the importance of Lck in the initiation of the proximal signaling events downstream of the TCR and consequently, T cell responses. It is therefore not surprising that the absence of Lck in both humans and murine models results in significant defects in immune functions whereas deregulation of Lck activity is often associated with cellular transformation. All these observations further emphasize the crucial role played by this kinase.

## LCK and coreceptors

Lck binds to the coreceptors CD4 and CD8 *via* a cytoplasmic “zinc clasp” formed by the double cysteine motif found in the coreceptor tails and the cysteines in Lck’s SH4 domain (22). The association of Lck with coreceptors is essential for coreceptor function as transgenic T cells or T cell hybridomas with truncated coreceptor tails, lacking the Lck binding domain, have diminished responses *in vitro* (23–31). How much Lck is physically bound to coreceptors is still debated and likely depends on the type of T cell studied (immature *vs*. mature, for example). Early studies using co-immunoprecipitation assays showed a significant fraction of cytoplasmic Lck bound to coreceptors (32, 33). However, more recent experiments showed much lower Lck to coreceptor occupancy, notably between 6 and 37% for CD4-Lck interactions in CD4+ single-positive (SP) cells (34, 35). Even lower occupancy values were found for CD8 SP cells and double-positive (DP) thymocytes. Targeting of Lck to membranes (plasma, vesicles, Golgi or ER membranes) is mediated by myristylation and palmitoylation modifications and preventing these modifications drastically impairs membrane targeting and TCR signaling (36). Consequently, an unknown amount of Lck is associated with plasma membranes versus internal membranes that do not contain coreceptors and these would appear in anti-coreceptor immunoprecipitates as “coreceptor-free’ Lck. As a result, immunoprecipitation experiments invariably under-estimate the true fraction of coreceptor-associated Lck in plasma membranes. Even so, the majority of Lck in immature double positive thymocytes is coreceptor-bound and that genetic knockdown of one the coreceptors leads to a dramatic increase in Lck associated with the remaining coreceptor (37). More precise biochemical or imaging techniques will be needed to settle the substantial discrepancies in Lck-coreceptor occupancy.

## Selection model

The selection model proposes that the delivery of Lck by the coreceptors during thymic development is the critical factor in imposing MHC restriction. Coreceptors play two fundamental roles; first, their specificity for invariant regions on MHC molecules allows tethering of the TCR to MHC, and second, their association with Lck allows the delivery of this kinase to the TCR-pMHC complexes to initiate signal transduction. Because all available Lck is bound to coreceptors in immature thymocytes, TCRs can only be signaled if they engage the same pMHC complexes as the coreceptors (CD4 for pMHCII-TCR complexes and CD8 for pMHCI-TCR complexes). TCRs that are specific for non-MHC ligands would not be signaled because Lck would not be recruited. Our model emphasizes that the sequestration of Lck away from the TCRs by the coreceptors ensures that only MHC-restricted TCRs can signal and be selected in the thymus (**Figure 1**).

## *In vivo* evidence of the selection model

To test the selection model, we generated several genetically manipulated mice. By disabling coreceptor-mediated Lck sequestration through germline deletion of both CD4 and CD8 coreceptors or by transgenic expression of a mutant Lck that cannot bind to coreceptors, we tested the hypothesis that non-MHC specific TCRs could signal in the thymus by “free” Lck and be positively selected to generate mature MHC-independent αβT cells. We called such mice Quad-KO mice since they are deficient for both CD4 and CD8 coreceptors and also lack MHC class I and II expression (38). Thymocytes in these mice were strongly signaled *in vivo* as shown by very high-level surface CD5 expression. Importantly we confirmed that MHC-independent signaling *in vivo* required the expression of αβTCR and Lck proteins as thymocytes deficient in TCRα, RAG2, pTα (unpublished data) and Lck showed reduced or absent CD5 upregulation (39). Furthermore, by forcing or preventing Lck sequestration through transgenic expression of wildtype or tailless CD4 proteins that encode either full-length CD4 or CD4 lacking the cytosolic tail, we confirmed that Lck sequestration significantly impairs TCR signaling in the absence of MHC (39). Importantly, deleting both coreceptors allowed the generation of mature αβT cells that were non-MHC specific (38). Notably, these TCRs had antibody-like properties in that they recognized conformational antigens with high affinity and in the absence of any antigen processing (40).

We have characterized in detail various MHC-independent αβTCRs isolated from Quad-KO mice. In our original studies, two of the Quad-KO TCRs recognized CD155, the mouse homolog of the poliovirus receptor, in its unprocessed form, independently of MHC and with affinities close to 200nM (40). These affinities are approximately 10- to 100-fold higher than conventional micromolar affinities of MHC-restricted TCRs (41, 42). As one of the TCRs we isolated used the same Vβ8 gene segments that contain germline-encoded residues and have been shown to contact MHC in crystal structures (43), we tested if the same residues were involved in non-MHC specific signaling and *in vivo* selection (39, 40). We found that the same germline-encoded CDR2 residues were also required for the thymic selection of the CD155-specific MHC-independent αβTCRs (39). These residues within the antigen-binding pocket are likely involved in contacting any protein, including, in this case, CD155. This result argues strongly against the model that these evolutionary conserved germline CDR residues enforce MHC binding (9, 44).

## Selection of MHC-independent αβTCRS

Our *in vivo* experiments showed that thymic signaling by CD155-specific αβTCRs occurred in the absence of any MHC and coreceptors, demonstrating the presence of αβTCRs that do not require MHC for their selection. Surprisingly, both CD155-specific TCRs absolutely required the presence of intra-thymic CD155 to signal thymic positive selection (39). These observations sharply contrast with conventional MHC-restricted αβTCRs, which require very low affinity ligand engagements for positive selection and for which very few selecting ligands have been identified (45–48). Our studies were the first to show a loss of function for a positively selecting ligand for any given TCRs that induce positive selection (39). Interestingly, using a series of mixed bone-marrow chimaeras, we demonstrated that the selection of mature CD155-specific αβT cells was achieved by all thymic elements (radio-resistant and radio-sensitive cells) and correlated with the amount of CD155 expressed (39). Ligands expressed on lymphoid elements in the thymus have been shown to select innate-like T cells, cells that can be characterized by the expression of the transcription factors PLZF for NKT cells or Sox13 for γδ-lineage T cells (49–51). None of our CD155-selected T cells expressed either PLZF or Sox13, confirming that CD155-specific peripheral T cells were neither innate-like NKT cells nor γδ-lineage T cells (39). As a matter of fact, thymocyte differentiation and lineage specification occurred normally in Quad-KO mice, as evidenced by CD4 reporter or TCR transgenic mice in which CD4 and CD8 αβT cells expressed the appropriate helper- and cytotoxic-lineage genes (38, 39). In our Quad-KO TCR transgenic mice, transgenic TCR expression occurs early at the DN stage and could have led to aberrant γδT cell differentiation. However, neither transgenic thymocytes nor peripheral Quad-KO αβT cells expressed specific γδT-lineage genes. Moreover, premature expression of a wildtype CD4 transgene, enabling CD4-mediated Lck sequestration at the DN stage, dramatically impaired positive selection (39). In summary, MHC-independent αβTCRs require *in vivo* expression of their cognate ligand for thymic selection, and they can be selected *in vivo* in the absence of coreceptors and MHC. This contrasts sharply with conventional MHC-restricted TCRs for which no defined *in vivo* ligands have been described to date and that MHC-restricted TCRs require coreceptor and MHC molecules for their selection.

## Diversity of MHC-independent αβTCRS

It was surprising that our first described MHC-independent αβTCRs were all specific for the same adhesion molecule CD155 and that both engaged CD155 with such high affinity. We therefore decided to test if CD155 was the only ligand for MHC-independent TCRs and if high affinity ligand engagement were a general feature of MHC-independent TCRs (52). Our first observation showed that Quad-KO mice that also lacked CD155 had the same number of peripheral MHC-independent αβT cells as did CD155-sufficient Quad-KO control mice, demonstrating that, *in vivo*, CD155 was not the sole thymic selecting ligand. We isolated and fully characterized additional Quad-KO TCRs that displayed high-affinity recognition of cell surface antigens CD155, CD102, and CD48. These native self-proteins normally function as low-affinity cell adhesion molecules. Like CD155 recognition, these newly isolated Quad-KO αβTCRs bind to and can be signaled by native unprocessed CD102 and CD48 in the absence of MHC (52). We used T-cell specific transgenic expression for one of those TCRs (specific to mouse CD102) and showed that this TCR signaled *in vivo* selection in the absence of coreceptors and MHC. Importantly, like the previously described CD155-specific TCRs, thymic positive selection required the expression of the native self-ligand CD102 (52). It was surprising to find that all the ligands identified for our MHC-independent TCRs were involved in cell adhesion. One reason could be that adhesion proteins are generally highly expressed on thymic cells (thymocytes and epithelial cells), increasing the likelihood of productive selecting signals (53, 54). Moreover, we have previously observed that some molecules including adhesion molecules like CD155 are downregulated during T-hybridoma fusions (unpublished data). Downregulation of these molecules impairs the fratricide of T-hybridomas expressing TCRs with those ligand specificities and allows their recovery during T-hybridoma fusions.

Other naturally occurring MHC-independent TCRs have been described over the years. These αβTCRs were also obtained from mature T cells but showed lower affinities (55–59). Because of their low affinity, it has been argued that their non-MHC ligands might not be their primary specificities (9). However, these TCRs were obtained from mature αβT cells that had undergone MHC-specific thymic selection and may cross-react incidentally with MHC-independent ligands. In Quad-KO mice, MHC-independent TCRs were signaled and selected by self-ligands with much higher affinity than those observed by conventional MHC-restricted TCRs.

The presence of high-affinity self-reactive αβTCRs in Quad-KO mice raises the possibility that signaling with free Lck prevents efficient clonal deletion. However, the reactivity of Quad-KO T cells selected in the presence or absence of the anti-apoptotic transgenic Bcl-2 (Bcl-2^Tg^) that is known to rescue deletion was identical to self and allogenic spleen stimulator cells. Irrespective of the transgenic Bcl-2^Tg^ expression, Quad-KO αβT cells were self-reactive as they proliferated in the presence of syngeneic (own Quad-KO) stimulator cells as well as against third party C57BL/6 and B10.A or BALB/c allogeneic splenic stimulator cells (52). We think that signaling by free Lck in the absence of coreceptor sequestration is inefficient in transducing high-affinity TCR signals to efficiently delete autoreactive thymocytes and prevent their emergence in the peripheral organs.

## Repertoire analysis of quad-KO T mice

Positive selection in the absence of MHC requires high-affinity TCR-ligand engagement, which could strongly affect the self-reactivity and diversity of the mature αβTCR repertoire. To test this hypothesis and learn what molecular constraints distinguish MHC-independent and MHC-restricted repertoire selection, TCR repertoire sequences in pre-selection thymocytes, mature MHC-restricted αβT cells, and MHC-independent αβT cells from Quad-KO mice were compared (60). Interestingly, we found that molecular constraints are imposed on hypervariable CDR3 segments during thymic selection of conventional MHC-selected repertoires. The length and amino acid composition of CDR3 segments were the primary parameters distinguishing both MHC-restricted and MHC-independent TCR repertoires (60). CDR3 lengths are known to vary greatly among αβTCRs, γδTCRs cells and immunoglobulins (61). Indeed, whereas CDR3s of both IgH and TCRδ are more variable in size and are longer than those in IgL and TCRγ chains, TCRα and TCRβ have almost identical CDR3 length, which is usually shorter than that of γδTCR and immunoglobulins. Interestingly, these differences are in accordance with their profoundly different recognition properties and requirements, both γδTCR and immunoglobulins functioning independently of MHC-and are, therefore, not constrained by the size of the MHC peptide binding groove. The conserved structure of peptide-MHC complexes limits the length of CDR3 on TCRs to favor shorter CDR3s, usually 8-13 amino-acids. TCRs with longer CDR3 structurally impair the contacts of their CDR1 and CDR2 with MHC. The position of the CDR3α and CDR3β at the center of the TCR-MHC contact interface requires the movement of the exterior CDR1 and CDR2 regions to accommodate longer CDR3s. In addition to peripheral MHC-independent TCRs, preselection thymocytes from normal MHC-expressing mice also contained TCRs with longer CDR3s, suggesting that MHC-dependent selection prevents the selection of TCRs with long CDR3s (60). Longer CDR3 could either signal MHC-specific clonal deletion or might fail to produce any MHC-specific signal and induce death by neglect. In fact, preventing clonal deletion in the thymus by introducing a Bcl-2 transgene did not result in the appearance of TCRs with longer CDR3 segments in the periphery MHC positive animals. Therefore, longer CDR3s does impair MHC binding and TCR signaling of MHC-specific positive selection in the thymus.

**Figure 2:**
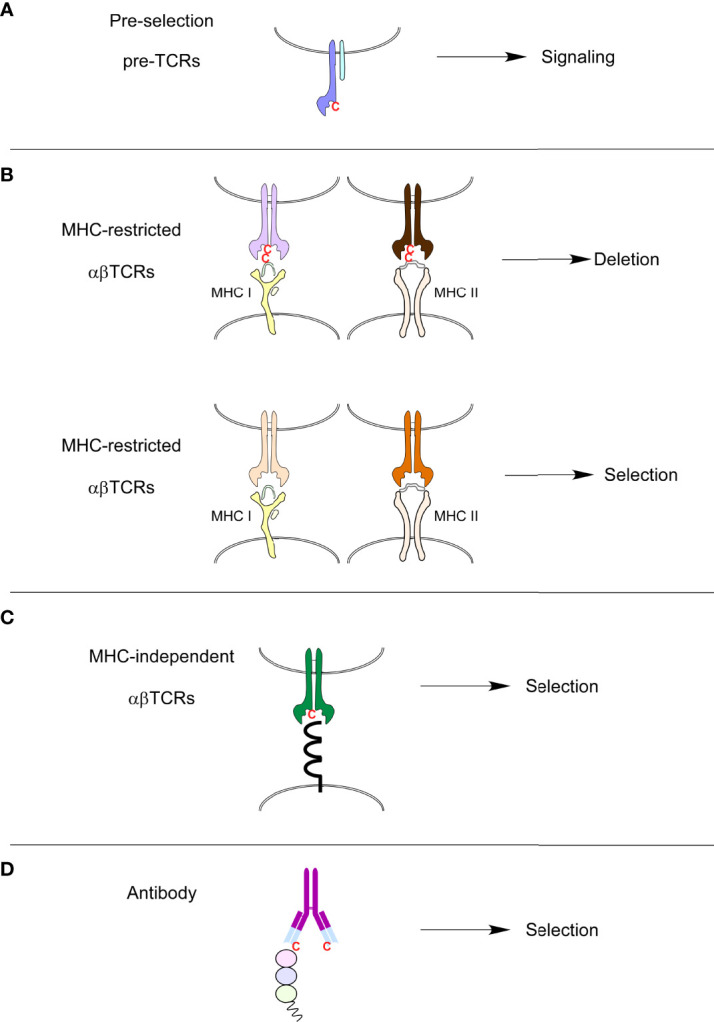
Presence or absence of cysteines in immune receptors. **(A)** Cysteines can be found in the FG loops of preselection TCRs. **(B)** MHC-restricted TCRs that contain cysteines in their FG loops are clonally deleted because cysteines will form disulfide-bonds with free cysteines in MHC-bound peptides present in the thymus. **(C)** MHC-independent ligands are extra-cellular proteins that very rarely possess free cysteines that could potentially link cysteines present in FG loops of MHC-independent TCRs. MHC-independent TCRs, just like antibodies **(D)**, do contain cysteines in their variable antigen-binding sites.

The usage of specific amino acids in CDR3s also puts some constraints on MHC-specific TCRs. For example, positively charged amino-acids (such as Lysine, Histidine or Arginine) were disfavored in CDR3 FGβ loops during MHC-restricted selection (60). The mature TCR repertoire is also controlled by clonal deletion, thereby eliminating TCRs with an excessive affinity for self-peptide/MHC ligands. Interestingly, we observed that clonal deletion during MHC-specific selection eliminated TCRs containing cysteines in their FG-loops (60). In fact, cysteines were present in 1-3% of TCRs in pre-selection and MHC-independent repertoires. Cysteines were, however, absent from mature MHC-restricted repertoires but were present in mice expressing the Bcl-2 transgene that prevents clonal deletion. Cysteines present in MHC-specific FG-loops would be crosslinked by MHC-presented peptides and induce clonal deletion (60). Interestingly, surface ligands recognized by MHC-independent TCRs do not contain free cysteines but rather have disulfide-linked cysteines (**Figure 2**). Such surface ligands would, therefore, not interact with the FG-loop cysteines from MHC-independent TCRs. Cysteines have a unique and critical role in protein function, structure, and stability. For extracellular and secreted proteins such as immunoglobulins, disulfide bonds formed between cysteine residues regulate protein scaffolding that allows proteins to maintain their three-dimensional structure (62, 63). Non-canonical cysteines can be found, although rarely, in immunoglobulins, even in the variable regions and are thought to participate in the generation of repertoire diversity (64) (**Figure 2**). Interestingly, Daley and colleagues found increased CDR3 cysteine usage in CD8αα intraepithelial T cells and their thymic precursors compared to regulatory T cells and conventional T cells (65). Thus, the presence of cysteine in the FG-loops serves as a TCR-intrinsic motif that could mark immature pre-selection thymocytes or some T cells with specific selection, such as the intraepithelial T cell or T cells selected by MHC-independent selection.

The comparison of TCR sequences from MHC-restricted and MHC-independent (both pre-selection cells and peripheral Quad-KO αβT cells) T cell populations allowed us to propose the important structural requirements of CDR3 for MHC-restricted and MHC-independent selection. The selection of MHC-independent TCRs seems to be largely unrestrained compared to the much more restrained selection of MHC-restricted TCRs. MHC restriction favors shorter than 13 amino-acids CDR3, prevents cysteine inclusion, and limits positively charged and hydrophobic amino acids in the CDR3β regions. The presence of conserved positively charged residues near CDR3β contact sites on both MHCI and MHCII molecules likely interferes with positively charged amino acids in the TCRβ sequences, inducing an electrostatic repulsion and preventing productive TCR-MHC interactions (60). Intriguingly, rare TCRs with longer CDR3α and multiple positively charged residues in CDR3β have been observed to bind MHC in a reversed orientation (66, 67). This reversed polarity could potentially be explained by the inability of the highly positively charged CDR3 FGβ loops and positive charges on MHC to form the canonical binding mode.

We also analyzed if thymic selection affected the usage of germline-encoded V- and J-genes and, their pairings. Interestingly, we observed similar frequencies of Vα-, Vβ-, Jα- and Jβ-genes between pre-selection, MHC-restricted and MHC-independent repertoires and animals of the same strain exhibited the highest similarities. The VJ pairing also showed similar frequencies among all groups (60). We concluded that neither V- and J-gene usages nor their pairing is significantly affected by thymic selection.

To assess the size of the TCR repertoire from Quad-KO mice, deep RNA sequencing of individual TCRα and TCRβ chains in Quad-KO mice was performed and compared with those from MHC-restricted wild-type strains. Importantly, wildtype mice had much greater repertoire overlap compared to Quad-KO mice. Common sequences were also shared among MHC-restricted strains but not among individual Quad-KO mice (60). We concluded that MHC-restricted repertoires show significantly higher sequence conservation than MHC-independent repertoires. The lack of shared sequences in the Quad-KO mice resembles that of antibody repertoires. Sequence diversities of the TCRα and TCRβ chains from MHC-restricted and MHC-independent TCRs were also analyzed. In fact, TCRs selected in the absence of MHC had dramatically lower (10- to 50-fold less) diversity compared to those from TCRs selected in the presence of MHC.

Overall, our repertoire analysis has shown that MHC-restriction severely constraints the length and composition of the hyper-variable CDR3 segments. In addition, positive selection by high affinity TCR-ligand interactions, such as those observed for MHC-independent TCRs, has dramatic effects on TCR repertoire diversity. Therefore, the presence of coreceptors during thymic selection permits the selection of a great variety of diverse TCRs with low affinity to self-peptide/MHC complexes.

## Structural analysis of MHC-independent αβTCRS

**Figure 3:**
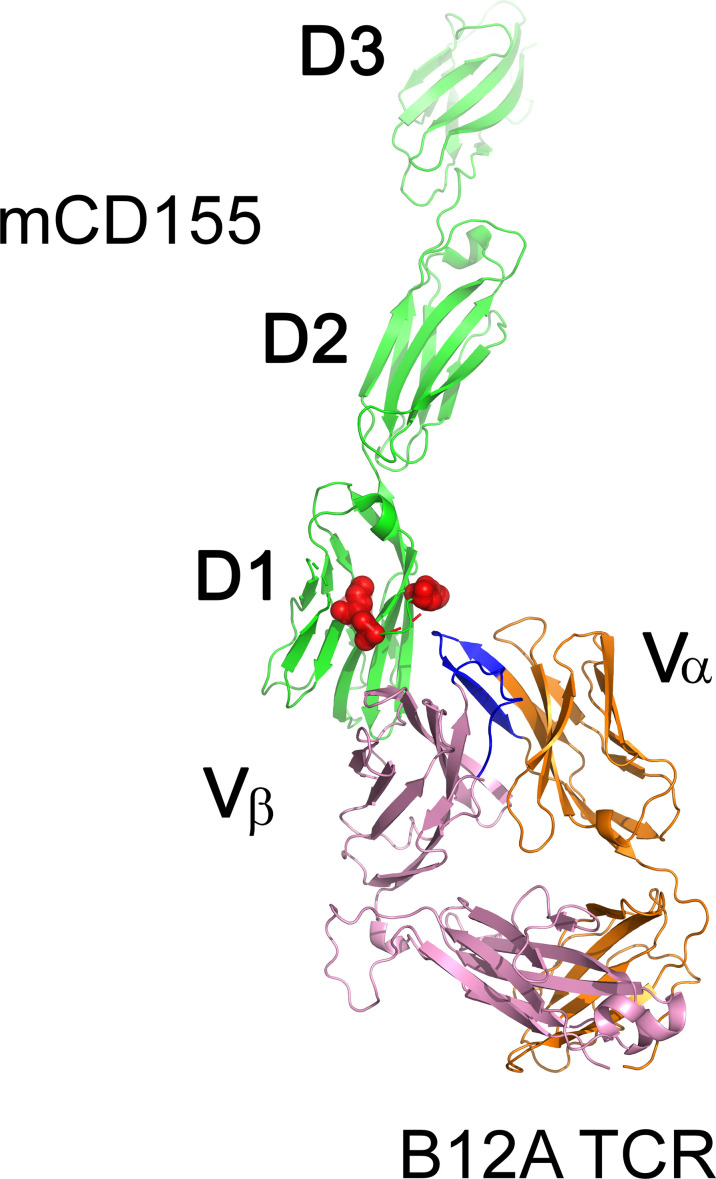
Surface representation of the MHC-independent B12A TCR with murine CD155. TCR B12A α β chains, and mCD155 are displayed as cartoons in orange, pink and green, respectively. CDR3 loops of TCR B12A are highlighted in blue and its binding epitopes on mCD155, revealed by mutagenesis studies, are highlighted as red surface.

To gain further insight into the biophysical properties of MHC-independent ligand interactions, we generated the first crystal structures of two MHC-independent αβTCRs and described their conformational epitopes on their ligand (CD155). Both TCRs (A11 and B12A) showed very high binding affinity to their CD155 ligand (230-280nM), values much higher than those typically observed for typical TCR-MHC binding (40, 68). A V-domain single chain of one TCR was sufficient to bind to CD155 with a 400nM binding affinity, a value only slightly lower than that of the two V-C domains. The B12A V-domain alone is, therefore, sufficient to recognize CD155. The structures of both αβTCR A11 and B12A αβTCR heterodimers were nearly identical and exhibited canonical structures when superimposed on the structure of an MHC-restricted TCR (68). Domain swapping experiment with murine and human CD155 revealed that both A11 and B12A TCRs required both D1 and D2 domains on CD155. This finding was confirmed using negative staining electron microscopy images of B12A TCR and murine CD155 and modeled in **Figure 3**. These images suggested that the CDR3s regions of B12A docked onto the D1 domain of CD155. Additionally, mutational experiments showed that A11 and B12A TCRs recognize two closely related but distinct epitopes on the D1 domain of CD155, a domain involved in binding to the poliovirus in humans (68). Interestingly, a third CD155-specific TCR (TCR 25) showed a different recognition motif (52), with domain swapping experiments for this TCR revealing that TCR-25 recognizes a novel epitope formed by all three external CD155 domains.

Structural experiments have provided evidence that some evolutionarily conserved residues (Y48 and E54) in the CDR2 region of Vβ8 TCR were necessary to engage MHC and impose MHC specificity on thymic selection (9, 43, 69). If these residues were conserved to engage MHC molecules, we would predict that such conserved CDR2 germline-encoded residues would not promote TCR selection by non-MHC ligands. However, we showed that the selection of an MHC-independent TCR containing Vβ8 also required the presence of the same conserved residues (39). These residues may have evolved for reasons unrelated to MHC binding but could possibly be involved in maintaining the integrity of the TCR combining site. It was also recently shown that an MHC-restricted TCR repertoire was still generated without the conserved germline-encoded CDR1 and CDR2 sequences (70). Dyson and colleagues replaced the TCRβ germline CDR1 and CDR2 regions with TCRγ chain CDRs (70). The resulting γβTCR hybrids paired with endogenous TCRα chains, provided efficient recognition of MHC and did not alter positive selection or CD4/CD8 lineage commitment. Receptors on γδT cells do not recognize MHC class I and II as natural ligands and, therefore, their germline encoded CDRs have not coevolved with MHC molecules. They concluded that T cell selection is not dependent on germline TCR structures and that the TCR can embrace antibody like strategies to engage MHC-peptide complexes. These observations were further confirmed by replacing the TCRβ germline CDRs with immunoglobulin (Ig) heavy and light chain germline CDRs, the resulting hybrid TCRs also led to the thymic selection of both CD4 and CD8 αβT cell repertoires (70). A novel population of naturally occurring T cells expressing a hybrid Vγ-Cβ TCR together with a TCRα has also been described (71). It suggests that the entire Vβ domain can be dispensable for MHC recognition. In summary, biophysical experiments have shown that, unlike conventional TCRs that only recognize peptide fragments complexed to MHC molecules, MHC-independent TCRs recognize a broad spectrum of conformational antigens. The combination of high-affinity binding and a variety of conformational antigens are typical characteristics of antibody recognition.

## Timing of coreceptor expression and LCK expression

Unconventional T cells such as mucosal-associated invariant T (MAIT) cells, natural killer T cells (NKT) and γδT cells are stimulated by lipid or metabolite antigens presented by monomorphic MHC-like molecules such as CD1 and MR1. Structural analyses have shown that the main characteristics of conventional TCR/MHC binding, namely the TCR conserved docking polarity of the TCR in which the TCR is placed over the peptide and simultaneously binds both MHC the peptide cargo, is also seen in unconventional TCR recognition of non-classical MHC molecules. Recognition of CD1 molecules, however, is inconsistent as some TCRs bind only to CD1 and not the lipid antigen. Interestingly, γδT cells, known to interact with ligands independently of MHC, have also been shown to interact with CD1d with a conserved polarity and docking angle.

**Figure 4:**
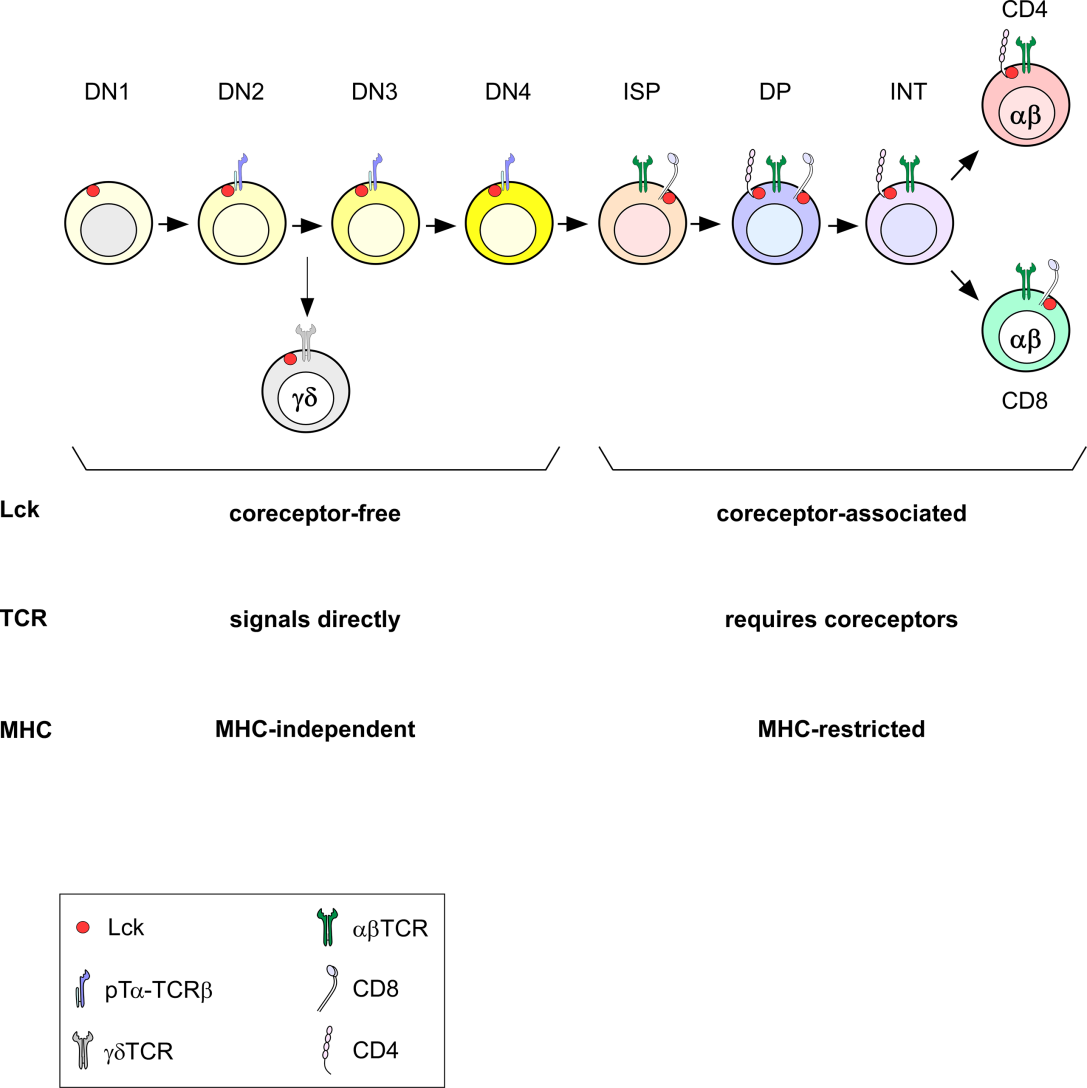
Timing of Lck expression during T cell development. During DN stages, Lck is necessarily coreceptor-independent (free). When coreceptors start being expressed at the DP stage, Lck becomes coreceptor-bound. In the absence of coreceptors at the DN stage, Lck can signal TCRs directly and signals independently of MHC (pre-TCR signaling at the DN to DP transition and during γδTCR selection). Once coreceptors sequester Lck, TCR signaling requires coreceptor engagement and is MHC-restricted.

As mentioned above, γδTCRs are mostly MHC-independent and are selected in the thymus before the DP stage (72–74). This early selection before the DP stage allows them access to free Lck (**Figure 4**). In normal conditions, all TCRs that are signaled and selected in the thymus before Lck sequestration by the coreceptors, such as γδTCRs, are MHC-independent. Early CD4 transgenic expression at the DN stage dramatically impairs the generation of γδT cells (39). We therefore think that the timing of endogenous γδ and αβTCR expression is precisely adjusted. This timing has evolved to permit different TCR complexes to selectively access either coreceptor-free or coreceptor-associated Lck so that ligand recognition by γδTCRs would be MHC-independent and ligand recognition by αβTCR would be MHC-restricted (**Figure 4**). Therefore, the appearance of coreceptors and the subsequent sequestration of Lck at the DP stage prevents positive selection signaling by MHC-independent ligands.

## Conclusions

A lot of information has been gathered since the first description of MHC restriction by Zinkernagel and Doherty more than 40 years ago (3). Recent experimental evidence supports both the germline-encoded and selection models, and both likely play a role in shaping an MHC-restricted TCR repertoire. The preselection repertoire may contain some proportion of MHC-biased TCRs but the requirement of Lck-coreceptor associations only permits and enhances the selection of a diverse but MHC-centric T cell repertoire. We think CD4 and CD8 play a central role in dictating the MHC specificity of the T cell repertoire and may have driven the co-evolution of αβTCRs with MHC. In other words, CD4 and CD8 coreceptors bestow the evolutionary pressure to skew germline TCR sequences toward MHC recognition. A better understanding of the biology of MHC-independent T cells will offer alternative therapeutic strategies, for example in immunotherapy. In conclusion, we think that MHC restriction of αβT cells is the consequence of thymic selection that imposes MHC-specificity by precisely timed expression of both CD4 and CD8 coreceptors on thymocytes.

## Author contributions

FL prepared the initial draft. AB, MC, JL, PS and AS revised and finalized the manuscript. All authors contributed to the article and approved the submitted version.

## Funding

The funding of this work is provided by the Intramural Research Program (IRP) of the US National Institutes of Health, National Cancer Institute, Center for Cancer Research.

## Conflict of interest

The authors declare that the research was conducted in the absence of any commercial or financial relationships that could be construed as a potential conflict of interest.

## Publisher’s note

All claims expressed in this article are solely those of the authors and do not necessarily represent those of their affiliated organizations, or those of the publisher, the editors and the reviewers. Any product that may be evaluated in this article, or claim that may be made by its manufacturer, is not guaranteed or endorsed by the publisher.
